# Structure Reversal of Online Public Opinion for the Heterogeneous Health Concerns under NIMBY Conflict Environmental Mass Events in China

**DOI:** 10.3390/healthcare8030324

**Published:** 2020-09-06

**Authors:** Jundong Hou, Tongyang Yu, Renbin Xiao

**Affiliations:** 1 School of Economics & Management, China University of Geosciences, Wuhan 430074, China; houjundong@cug.edu.cn; 2 School of Management, South-central University for Nationalities, Wuhan 430074, China; ytyd535@126.com; 3 School of Artificial Intelligence and Automation, Huazhong University of Science and Technology, Wuhan 430074, China

**Keywords:** online public opinion structure reversal, heterogeneous health concerns, agent-based model, NIMBY conflict

## Abstract

Public opinions play an important role in the formation of Not in My Back Yard (NIMBY) conflict environmental mass events. Due to the continual interactions between affected groups and the corresponding government responses surrounding the public interests related to health, online public opinion structure reversal arises frequently in NIMBY conflict events, which pose a serious threat to social public security. To explore the underlying mechanism, this paper introduces an improved dynamic model which considers multiple heterogeneities in health concerns and social power of individuals and in government’s ability. The experimental results indicate that the proposed model can provide an accurate description of the entire process of online public opinion structure reversal in NIMBY conflict environmental mass incidents on the Internet. In particular, the proportion of the individual agents without health interest appeals will delay the online public opinion structure reversal, and the upper threshold remains within regulatory limits from 0.4 to 0.5. Unlike some previous results that show that the guiding powers of the opinion leaders varied over its ratio in a fixed-sized group, our results suggest that the impact of opinion leaders is of no significant difference for the time of structure reversal after it increased to about 6%. Furthermore, a double threshold effect of online structure reversal during the government’s response process was observed. The findings are beneficial for understanding and explaining the process of online public opinion structure reversal in NIMBY conflict environmental mass incidents, and provides theoretical and practical implications for guiding public or personal health opinions on the Internet and for a governments’ effective response to them.

## 1. Introduction

Currently, although a larger number of urban public facilities advocated by the governmental agents can benefit from the development of cities as a whole, e.g., the nuclear power plants and waste infrastructures, the proximate residents usually oppose and resist them once they believe that their living environmental and personal health are compromised, easily causing environmental mass events [1]. Essentially, this is the Not in My Back Yard (NIMBY) conflict phenomenon [2], which usually induces large-scale collective activities for common interests surrounding human health in the interaction among cyber and actual individuals [3]. It is not unusual that public conflicts are derived from NIMBY events, and numerous studies focus on their social impacts around the world [1]. Due to the large population of netizens and small-world features possessed by social networks [4], e.g., Weibo, Wechat, Twitter and Facebook, the affected groups usually tend to employ it to intensely propagate public opinions on this type of events. This is because the traits of social contradiction would not be the same to that in the online society as the capabilities regarding discourse power, information acquisition and resonance of ordinary citizens have been enhanced by social community [3]. From this background, it is easy to present a “network structure reversal” effect that relies on NIMBY facility development as a carrier [5].

As a special and novel phenomenon in the evolution of the social system, the intrinsic nature of online public opinion structure reversal is the process that the initial attitude of a mighty government (minorities) gradually evolves and updates to be the same as the opinions of the disadvantaged individuals (majorities) at a steady state. In addition, its external manifestation is the reversal of the group power structure in the real and online society, i.e., traditional bodies with strong authorities (i.e., governments) are in a relatively weak position on the network, whereas the counterparts with weak power (i.e., residents) become very strong. For example, in the para-xylene (PX) chemical project or waste incineration power generation project, there is a typical phenomenon of mass incident network reversal of public opinion that the interest appeals of disadvantaged citizens were accepted into the public policy agenda while the corresponding upper level governments were compromised. According to Homans’ theory of collective behavior [6], groups as social systems are made up of two subsystems—the external and the internal; thus, generally we consider that online public opinion structure reversal may mainly take place in the event space where network and reality are intertwined, focusing on the sensitive issues of public decision regarding environmental health. If the dissemination rules of public opinion in social media fail to be explained, in certain situations it will not only lead to negative consequences for social stability [7], but also result in a crisis of trust in the governments [3]. Therefore, it was observed that exploring the evolutionary process and law of public opinion of NIMBY conflict mass event can importantly contribute to our understanding of its predicting and warning, as well as governments’ response [8], so this topic has attracted considerable interests from researchers in different scientific fields [9,10,11,12].

Evolution of public opinion is an extremely complex process [13]. Moreover, the public’s initiative, spontaneity and community in social media will be affected by individuals and environmental factors [10]. Building a reasonable model to map the evolutionary process of public opinion can improve our support of the elucidation of its intrinsic mechanism. In general, the cognition process absolutely plays a central role in individual decision-making. As we know, because participants with different features have some differences in health cognition of events, the decision-making of participants in mass incidents will also not be the same, while the consistency of decision-making is crucial to the network public opinion structure reversal. Although prior studies proposed heterogeneous interaction models [7], most of them were the basic dynamic models that could describe the traditional diffusion of public opinion from the individual level. Nevertheless, the significance of interactions between individual and government agencies cannot be overlooked [14]. Accordingly, this research seeks to propose an improved model to investigate the structure reversal of online public opinions of NIMBY facility development considering multiple heterogeneities of individual health concerns and government responses simultaneously.

This study contributes to the insight implications in two aspects. Firstly, we put particular emphasis on the interplay role of individual and governmental heterogeneity in the reversal process, because they are critical determinants in collective actions. Secondly, the combination of new factors including individual interest appeal to personal health and public authority, and governmental response capacity is expressed quantitatively to describe the interactive process. Therefore, findings will be useful to improve the public engagement in policy agenda setting, and intervention and prohibition of social conflict.

The rest of this paper is organized as follows. We begin with a review of related works in Section 2, and point out their corresponding limitations. Section 3 puts forward a leader-follower opinion dynamic model considering multiple heterogeneities about individual interest appeal on health concern and influence power, and government response capacity based on the brief description of heterogeneity regarding different agents in the mass event. The following section is the research framework of this paper. In Section 5, we report and discuss some computer simulation results on the structural reversal of public opinion. We conclude this study and give some potential research directions in Section 6.

## 2. Related Works

Public opinion is the collective attitude of citizens on a given government policy or issue, and the public’s attitudes can vary over time [15]. The pressure of public opinion is a source of constant conflict for governments in weighing measures in order to win public support [16]. Under this circumstance, to prevent the potential conflict, how to apply a dynamic model to capture the essence of formation and evolution of opinions within a group of members has been a subject of interest in many fields. In the early research stage, some scholars argued that it could be described and investigated based on the infectious disease model [17] and the rumor propagation model [18] with the reference of the Susceptible Infected Recovered model (SIR) [19] to analyze this issue. According to the high uncertainty of a group’s opinion, and the different results of consensus, polarization, and diversity of being appeared at certain stages, researchers had proposed some classic models for opinion dynamics. In fact, considering the domain of the opinion, previous works could be categorized into discrete and continuous opinion dynamics models. The former included Sznajd model [20], Voting model [21], Ising model [22], etc. However, it has been found that discrete model was difficult to describe the continuous transitions in public opinions, thus the idea of continuous value (i.e., a number between 0 and 1) capturing an individual opinion was applied in modeling of public opinion. For example, Krause and Hegselmann [23,24], and Deffuant and Weisbuch [25,26] respectively proposed the continuous public opinion dynamics models based on bounded confidence level, namely the Krause–Hegselmann (HK) model and Deffuant–Weisbuch (DW) model. Subsequently, these two models received great attention in the field of opinion dynamics, and improved and extended for the specific phenomena and problems in order to reasonably evolve the public opinion. The improvements based on the DW model mainly focused on reciprocity feedback consistency [27], first impression effect under general opinion distributions [28], interaction selection rules [29], steady-state property in social networks [30], social learning with heterogeneous agents [31] dynamics of bounded confidence threshold [32], noise impact [33], interpersonal network [34]. The expansions of HK model were reflected in confidence threshold [35], nonlinear viewpoint updating rules [36], opinion leaders [37], trust threshold heterogeneity [38], directed network [39], self-confidence parameter [40], cognitive styles [41], etc. Additionally, some studies discussed related approaches to the examined topics to investigates users’ communities, influence spreading and recommendations based on social media networks [42,43,44].

Some authors started to conduct studies regarding public opinion reversal. For example, Chen et al. [12] proposed a model to identify individual internal characteristics and external intervention information that affect the reversal; Hou and Hu [45] considered information as a variable and embedded it into bounded confidence model to explore the impact of information on public opinion reversal; Xiao et al. [46] established a novel model which takes the effects of natural reversal parameter into account based on the HK bounded confidence model. Obviously, previous research on public opinion reversal still aimed to reveal the formation and evolution processes, rather than the essence of “structural reversal effect” in online society emphasized in our study.

In short, the prior works mentioned above could provide us with a more in depth understanding of processes and results of individual opinions through online interactions, obviously in real social systems; however, there are some gaps to be solved yet: (1) Since the update of an individual’s opinion will be the result caused by the combination roles of internal psychological factors and external social influences [46], the evolutionary process will be much more complex, and there is often inconsistencies or reverse transformation in the public opinion expression between the early and late stages of event. Thus, an improved opinion dynamics model was advanced for understanding the evolution laws of reversal of online public opinion from the perspective of statistical physics [9,47]. (2) In the online public opinion structure reversal of NIMBY conflict event, the participants impaired from a policy were less and less satisfied with this imposed distribution pattern. Thus, in the process of using the protest to express interest demands about health and to achieve reasonable and rational gains, the diversified interactions become inevitable between the government benefiting from the policy and the social public impaired with this policy. Although some literature had examined policy responsiveness to the public and to interest groups separately, studies of public policy that integrated both factors were limited [14]. Besides, the traditional dynamic models described the interactions between individuals in real life, as well focusing on the general public opinion diffusion under linear or nonlinear rules [24], but there still remains no work that systematically considers the government response capacity on the role of network public opinion. (3) It is not unusual that the concerns about human health are derived from NIMBY facilities [1]. On one hand, interest attribute of the agents could be roughly divided into two categories: the individuals with (i.e., policy losers) or without interests (i.e., bystanders) regarding health issues. Their different interest appeals might result in different processes of opinion interaction and evolution in this event. On the other hand, the agents also could be categorized into another two ways by the influence power: ordinary individual or opinion leader. It is clear that most studies indicated that opinion leaders played an important role in information propagation. In many cases, opinion leaders may transfer the information to the neighboring agents unconsciously [48]. However, in some NIMBY conflict cases, opinion leaders hoped to guide the neighbors to an expected opinion for a purpose, such as panic boycott. It indicated that the behavior of agents was different from that in the existing literature. Accordingly, the previous models failed to explain these heterogeneities and were difficult to accurately analyze these new conditions. Therefore, in this paper an improved model is proposed which introduces interest appeal in health concerns and social power of individuals to reflect the influence of individual internal characteristics on their attitudes, as well introduces the heterogeneous abilities of the government agency to depict the effect of external intervention on individual opinions, so as to explore the process of online public opinion structure reversal in mass incidents using the agent-based method.

## 3. Proposed Dynamics Model Considering Multiple Heterogeneities in Health Concerns

In general, individuals, communities, and governments are the categories of social media users [49]. Among them, communities include groups of persons who share expertise, values, norms, interests, and experiences [50], and are further divided more specifically into celebrities and journalists, namely opinion leaders. Thus, this paper defines the participating agents in the diffusion network as individuals, opinion leaders and the government.

### 3.1. Individual Heterogeneity

**Figure 1:**
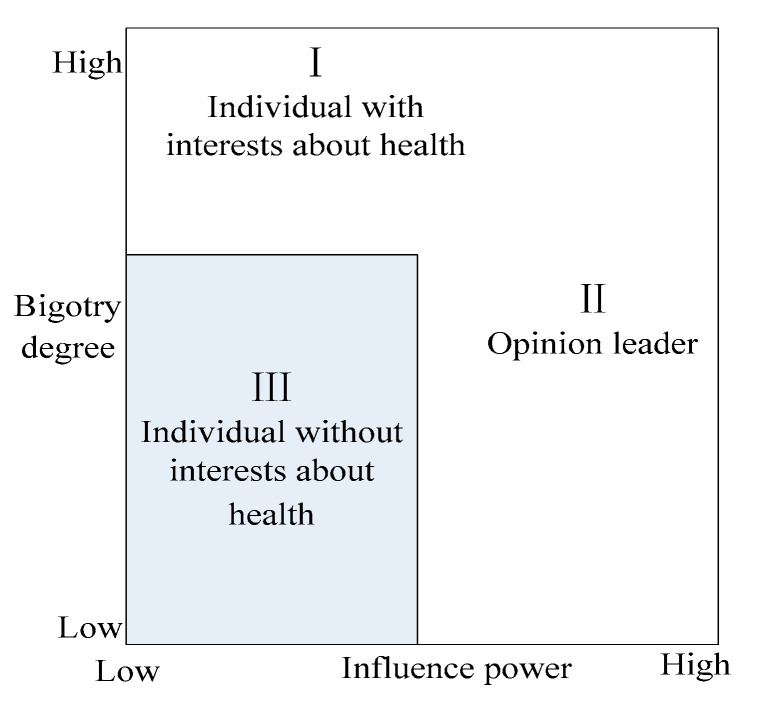
Heterogeneity of individual agents.

The non-consensual opinions of different individuals seem to be a hotbed of social problems, and a key driving force for the evolution of NIMBY environmental conflict event as well. It can be observed that different individuals tend to exhibit different social attributes and updating ways of opinions [46]. For this reason, we segmented the individual agents from the perspectives of interest appeal about personal health and influence power (as shown in Figure 1). Herein, interest appeal about personal health will affect the bigotry degree of individual agents involved in online public opinion, and influence power will determine their authority and influence scope.

#### 3.1.1. Heterogeneous Interest Appeal Regarding Personal Health of Participants

Normally, the degree of health interests damaged is positively related to the possibility of protest [51]. In the public opinion process of NIMBY environmental conflict events, participants interact with and infect each other. They usually can be divided into two different types of groups: the individual with direct or indirect health interests (i.e., affected groups), and the bystander without any interests (i.e., unaffected groups), respectively. The motivation of agents with interests is mainly to express their appeals and attitudes regarding NIMBY facility, or directly seek and safeguard their own interests, while the bystanders mostly bring about the emotional resonance to take part in the event affected by the herd effect in the irrational behaviors. So we can safely deduce that the proportion of affected groups and unaffected groups participating a given incident will have an impact on public opinion structural reversal. In contrast, if the ratio of participants without interests is higher, the probability of network public opinion structure reversal might be lower.

Firstly, let *A* be a set of *n* nodes, and a *n* × *n* matrix *R* is to represent the relationship matrix between individuals in a social network. For all individuals *i* and *j* in set *A*, *R_ij_* means whether individual *i* is connected with *j*. When *R_ij_* = 0, it denotes individual *i* is not connected with *j*; and when *R_ij_* = 1, there is a link between them. Then, supposing the amount of agent with interest in a network is *m*. Thus, the number of agents without interest is *n* − *m*. After that *p*arameter *hetero* is assigned to denote this ratio, and:

*Hetero* = (*n* − *m*)/*n*. (1)

Generally, *hetero* is a constant within the interval [0, 1]. Nevertheless, extremists in any activities only account for a small portion due to the 80/20 rule proposed by Italian economist Pareto [8]. In our simulation model, given *hetero* = 0.2 as a default value for the sake of simplicity, the rest of the 80% are those individuals with interest appeals.

#### 3.1.2. Heterogeneous Social Influence of Participants

Based on the social influence of different individuals, the participants can be divided into two categories: opinion leaders and ordinary individuals. Generally, the influencing levels of these two kinds of agents’ opinions on an individual’s attitude seem to be not the same within the network.

Opinion leaders are usually considered to be advanced influential users, and capable of having a profound impact on the opinion formation of other users (especially ordinary participants) in the propagation process of public opinion [46]. It is not similar to unconsciously transfer the information to the neighboring agents, by virtue of their authoritativeness and a certain popularity, the opinion leaders with a large number of followers in the NIMBY conflict cases cannot just share their views and related information to the common Internet users through web filtering approach, but also motivate the majority of other agents and thus stimulate community movements so as to realize the purpose of agenda setting. This presents a phenomenon that opinion leaders can consciously guide the adjacent individuals to achieve expected opinions. However, opinion leaders with higher influence are more likely to promote the interactions of individual views. Therefore, according to the dynamics model with the bounded confidence, this study applies confidence threshold *d* (that is, the opinion distance between two individuals) to distinguish the heterogeneity of social influential degree between the two agents. The smaller the value of *d* is, the easier it is for individuals to interact with each other. It means the confidence interval of opinion leaders is usually smaller than that of ordinary individuals [17]. In addition, public opinion in a social network with opinion leaders tends to spread much faster than that without opinion leaders [52]. This indicates that opinion leaders will affect the propagation speed of event, which in turn plays a role in the public opinion structure reversal of mass event. Although the number of opinion leaders is not unlimited in any given social network, there are at least two subgroups with positive and negative target opinions, respectively. Consequently, it is not a truth that the greater the number of opinion leaders, the better the performance of diffusion. Thus, parameter *nLeader* is measured as the total number of opinion leaders during the structure reversal of network public opinion.

For the convenience of description, *nLeader* is a constant. Based on reference [53], diffusion may occur only when the amount of adoption is beyond a certain rate, and it is usually about 10% of the population. Therefore, opinion leaders account for 10% of the total number of participants, this is regarded as a default in the simulation experiment. Theoretically, opinion leaders are either agents without or with an interest. In reality, opinion leaders can be experts or be very interested in a subject (e.g., celebrities and journalists), and they are noted for their interventions on specific topics, rather than for their interest appeals.

### 3.2. Governmental Heterogeneity

The government’s actions in response to NIMBY conflict cases are selective and different [51]. In the different interactive stages between government agencies and the individuals, the corresponding response states are usually not identical. However, in order to simplify the analysis process, our research assumes that there are only two response states in each interaction process. One is pressure-response state determined whether the corresponding government is necessary to intervene, and the other is compromise-consensus state which depends on its regulatory ability for the diffusion of an event. Obviously, there are heterogeneities for the different governments, all of which will play a clear role in the evolution of public opinion.

#### 3.2.1. Heterogenous Pressure-response Ability of the Government

With promoting the modernization of state governance, the government’s response to online public opinion is an important criterion that reflects its ability and level of decision-making, whereas passive pressure-response has become the main way in which local government deals with online public opinion at present. Actually, considering a series of influencing factors, such as pressures from upper levels of government and affected groups, their own value preferences, expert knowledge and public opinion, the government usually prefers to intervene (e.g., official announcements) when the public opinion risk becomes a substantive realistic social impact, which is often a critical point for the government to take countermeasures in the NIMBY case. Thus, parameter *pWarning* is assigned to measure this heterogeneity of pressure-response ability of the government in a NIMBY mass event, which is a constant within the interval [0, 1]. The higher this value, the stronger its intervention ability.

#### 3.2.2. Heterogeneous Compromise-consensus Capability of the Government

The magnitude of a government’s ability deposing the social conflict incident is dependent on its determination to promoting a certain decision, resources endowment, and emergency system, etc. During the period of public opinion that the government deals with it negatively and the public vigorously contest, once the evolutionary trend is out of control, the government will be forced to compromise and reset public issues into policy agenda based on the idea regarding social stability maintenance. In other words, the government will shift its initial attitude or decision on NIMBY facility development to meet the individuals’ interest appeals and build a consensus, which is the final solution to the government’s passive response to a focus on an incident changing from a “pressure-response” state to a “consensus-building” model. However, the heterogeneity of comprehensive intervention capability formed by the government’s response capability and risk-resistance level will impact this critical turning point in the online public opinion structure reversal of NIMBY environmental incidents. Accordingly, parameter *pJustice* is applied to measure this heterogeneity, and which is a constant within the interval [0, 1]. The larger this value, the stronger its resist-risk ability.

### 3.3. Dynamics Model of Opinion Update

In the finite set *A*, *x_i_* depicts the attitude value of agent regarding NIMBY facility development. Thus, the opinion at time *t* of agent *i* is denoted by *x_i_*(*t*) ϵ [0, 1] (*i* = 1, ....., *n*). Initially, *x_i_* (*0*) is assigned a continuous random uniform opinion value in the range [0, 1], where 0 and 1 stands for extreme agreement and disagreement, respectively. In addition, when *x_i_* (*t*) ϵ (0.5, 1], the individual *i* tends to oppose or have distaste for the NIMBY facility in question. Otherwise, he or she is inclined to favor it.

At each discrete time *t*, each agent interacts with other connected agents, and their interaction rules are conducted based on bounded confidence. However, due to the different interest appeals and influential degrees of individuals involved in a NIMBY event, their opinion update processes should not be the same. Therefore, we now propose our novel dynamics models for the public opinion of agents based on the heterogeneous attributes, respectively.

#### 3.3.1. Opinion Interaction Modeling between Ordinary Individuals

If both agents *i* and *j* are ordinary individuals. Traditionally, agent *i* is impacted by its neighboring node *j*, while this agent may be either a participant with interests or the one without interests from the health perspective. Thus, there are some cases as follows.

**Case 1**. If both agents *i* and *j* are ones without health interests, for any NIMBY cases, because these agents do not involve the expression of interests, at the initial stage they usually do not pay more attention to public opinion of the incident in focus, but the network structure in which the individual is located (i.e., the neighbor node) often impacts his/her diffusion attitude. Therefore, the opinion of an agent *i* might change as it gets influenced by its neighbor’s opinion in a given social network. Thus, an opinion updating model is proposed for the agent without an interest at every time *t* as Equation (2).

(2) $$x_{i}\left(t+1\right)=\overset{n}{\underset{j=1}{\sum }}a_{ij}x_{j}\left(t\right)$$

where *x_i_*(*t*) is attitude value of agent *i* at time *t*, and *x_j_*(*t*) denotes the attitude of the *j^th^* neighbor adjacent to agent *i.* The coefficient *a_ij_* represents the impact that agent *i* exerts on agent *j*, its value is the results of normalizing the degree of agent *i*, it is computed by Equation (3).

(3) aij={jdegree∑l=1kldegree, if Rij=10, if Rij=0

where *j_degree_* is the degree of agent *j* connected with agent *i*, *l_degree_* is the degree of a node *l* connected with agent *i*; $\sum_{j=1}^{k}a_{ij}=1\;$; *k* is the degree of agent *i*, which means the number of neighbors of agent *i.*

**Case 2**. If both agents *i* and *j* are the ordinary agents with interest in public health in the NIMBY event, in order to express their interests and set policy agendas, the affected residents often resort to stress-responsive actions (e.g., “protest with death”) or strategic actions (e.g., “performance-based protest”) to trigger the issue of radical petition [3]. In this process, undoubtedly the participants with interest usually pay close attention to public opinion of the NIMBY mass incident, and it is not appropriate to adopt the attitude updating method of average opinion in the original dynamics model. Generally, in the condition of information asymmetry, public opinion among the agents with interest appeals in a mass incident is featured in bounded confidence [25]. That is to say, the difference in opinion between two neighbors is not greater than a specific confidence threshold, they can share or influence one another, incrementally changing opinions to become more similar to each other. Therefore, it is suitable to employ the bounded confidence DW model to accurately describe the propagation process of public opinion for the agents with interests. The equations are as follows.

(4) $$x_{i}\left(t+1\right)=\{\begin{array}{c} x_{i}\left(t\right)+\mu_{i}\left[x_{j}\left(t\right)-x_{i}\left(t\right)\right],\left|x_{j}\left(t\right)-x_{i}\left(t\right)\right|\leq d_{1}\\ x_{i}\left(t\right),\left|x_{j}\left(t\right)-x_{i}\left(t\right)\right|>d_{1} \end{array}$$

(5) $$x_{j}\left(t+1\right)=\{\begin{array}{c} x_{j}\left(t\right)+\mu_{j}\left[x_{i}\left(t\right)-x_{j}\left(t\right)\right],\left|x_{j}\left(t\right)-x_{i}\left(t\right)\right|\leq d_{1}\\ x_{j}\left(t\right),\;\left|x_{j}\left(t\right)-x_{i}\left(t\right)\right|>d_{1} \end{array}$$

where *x_i_*(*t*) and *x_j_*(*t*) are opinion values of agent *i* and *j*, respectively. *d*_1_ represents the confidence threshold for the interaction between ordinary agents.

In addition, although influence power of affected groups is not high, their tendency of public opinion on the event is rather bigotry, which will accelerate the spread and update of views until it converges. To distinguish the heterogeneity of bigotry degree caused by different interest appeals of agents, the convergence parameter *μ* is introduced in our study. The smaller the *μ*, the more likely the user is inclined to hold his/her initial opinion, and vice versa. This also indicates that the convergence coefficient of the stakeholders is lower than that of the participants without interests. Generally, *μ* is constant within the interval (0, 0.5].

**Case 3**. If agent *i* is an ordinary individual without health interests in NIMBY mass event, while agent *j* is the one with interests. In the real world scenario, an individual is not affected by another node if their opinions differ greatly. However, as the agents with health interests are more bigotry about their own views and have a certain incitement and contagion effect on the agents without interests, agent *i* is somehow difficult to ignore agent *j*’s opinion even if the opinion difference between agent *i* and agent *j* is higher than the threshold. Considering the cognitive judgment of agent *i*, in this case agent *i* will accept agent *j*’s opinion with a certain probability as shown in Equation (6).

(6) $$\{\begin{array}{c} P\left[x_{i}\left(t+1\right)=x_{j}\left(t\right)\right]=p\;\\ P\left[x_{i}\left(t+1\right)=x_{i}\left(t\right)\right]=1-p \end{array}$$

where *p* is assigned a random uniform opinion value in the range [0, 1].

However, as for the agent *j*, even if the agent *i* is an agent without interests, as long as $\left|x_{j}\left(t\right)-x_{i}\left(t\right)\right|\leq d_{i}$, the opinion of agent *j* will still be updated according to formula (4). Otherwise, agent *j’s* opinion will not be changed.

#### 3.3.2. Opinion Interaction Modeling between Opinion Leaders and Individuals

As analyzed above, most of opinion leaders are agents without an interest. Besides, during the interactive process, the influence differences of opinion leaders with and without interests can be weighed by their different initial attitude values. Thus, to simplify the simulation experiment, supposing agent *i* and *j* are ordinary individual and opinion leader, respectively. There are some cases for the evolution of agent *i*’s opinion.

**Case 4**. If agent *i* without interest interacts with an opinion leader agent *j*, due to the stronger influence of opinion leader at the overall network scale, agent *i* is difficult to ignore agent *j*’s influence and will passively accept the attitude of agent *j* who is an infected source. The opinion update modeling for agent *i* is Equation (7), while the opinion value of agent *j* remains unchanged as an opinion leader has unambiguous objective opinion [54].

(7) $$x_{i}\left(t+1\right)=x_{j}\left(t\right)$$

**Case 5**. If agent *i* with interest interacts with an opinion leader agent *j*, both agent *i*’ and *j*’s opinion updating processes will strengthen *μ* based on the formulas (4) and (5), such as let it increase with a random number *ε* as shown in Equations (8) and (9).

(8) $$x_{i}\left(t+1\right)=\{\begin{array}{c} x_{i}\left(t\right)+(\mu_{i}+\varepsilon )\left[x_{j}\left(t\right)-x_{i}\left(t\right)\right],\left|x_{j}\left(t\right)-x_{i}\left(t\right)\right|\leq d_{2}\\ x_{i}\left(t\right),\;\left|x_{j}\left(t\right)-x_{i}\left(t\right)\right|>d_{2} \end{array}$$

(9) $$x_{j}\left(t+1\right)=\{\begin{array}{c} x_{j}\left(t\right)+(\mu_{j}+\varepsilon )\left[x_{i}\left(t\right)-x_{j}\left(t\right)\right],\left|x_{j}\left(t\right)-x_{i}\left(t\right)\right|\leq d_{2}\\ x_{j}\left(t\right),\left|x_{j}\left(t\right)-x_{i}\left(t\right)\right|>d_{2} \end{array}$$

where *ε* is a random value generated between (0, 0.5], and *d*_2_ depicts the confidence threshold for the interaction between ordinary agent and opinion leader.

In addition, it needs to be noted that when agent *i* and *j* are connected, their interaction is considered. Otherwise, the agents will keep initial opinions. Besides, the opinion of individuals does not continue to change, or the following condition in Equation (10) is met, the update process will be ended.

(10) $$\overset{n}{\underset{i=1}{\sum }}{\left[x_{i}\left(t+1\right)-x_{i}\left(t\right)\right]}^{2}\leq \delta$$

where *δ* is a positive value close to 0, which is set to 0.000001 in this paper.

#### 3.3.3. Opinion Interaction Modeling between the Government and Individuals

Some literature investigated the extent to which public opinion is related to public policy design [5], as well as the role of interest groups, while the question of how this individual group influence really is remains unsettled [14]. In this research, the government agency is regarded as an exogenous influencing factor in the public opinion dissemination process. When the opinion at time *t* of a government is denoted by *y(t)*, and *y* ϵ [0, 1], the opinion of government agency completely supporting or opposing the NIMBY facility is defined by *y(t)* = 1 or *y(t)* = 0, respectively. As discussed before, *pWarning* and *pJustice* are assigned as two thresholds for the government’s response to the NIMBY event, and *pWarning* is often less than *pJustice* in real world. Thus, the interaction rules between the government and individuals are shown in these two cases below.

**Case 6**. If *b* > *pWarning*, the corresponding government start to take measures (e.g., official announcements, mandatory regulation, public hearing) to regulate public opinion of NIMBY conflict mass incident.

(11) b=∑1nxi(t)n 

where *b* is defined as the average opinion of all individuals.

**Case 7**. If *b* > *pJustice*, the corresponding government will shift its own attitude from *y(t)* = 1 to *y(t)* = 0, vice versa, which represents the structure reversal of public opinion to NIMBY mass incident in our study.

## 4. Research Framework

Agent-based model (ABM) is a class of computational models for simulating the actions and interactions of autonomous agents (both individual or collective entities such as organizations or groups) with a view to assessing their effects on the system as a whole [55]. Based on the proposed dynamics models, a computer simulation method of agent-based model is adopted to investigate the effect of multiple heterogeneities on structure reversal of public opinion of mass incident.

### 4.1. Simulation Scenario and Experimental Design

**Figure 2:**
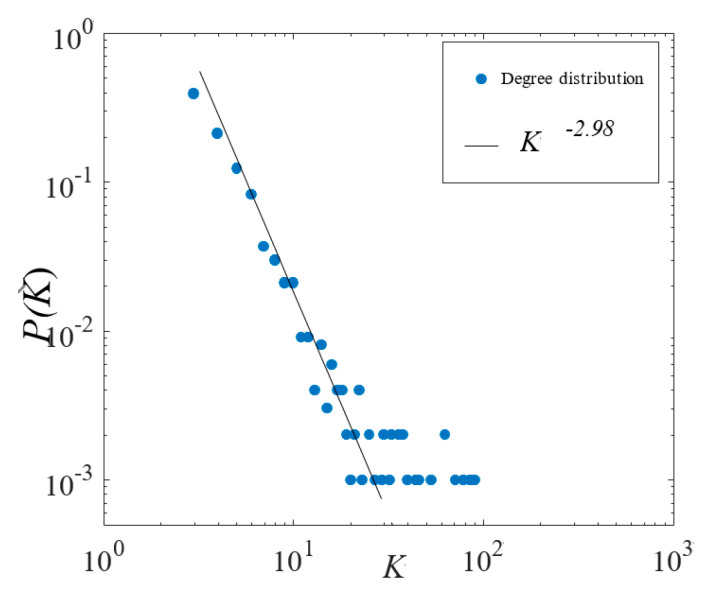
The log-log plot of degree distribution in Maoming PX event.

In the NIMBY conflict network, the participants are mostly residents in the incident-affected area. Compared with the total number of nodes or connections, in the network formed by these agents, the shortest paths of any two nodes are very small, and its scale distribution of connected sub-graphs is a typical of power law distribution (Figure 2). Besides, since there are complex social network structures in the mass incidents, all of them will be shifted from the simple random network to the complex non-random network [18]. Accordingly, this paper only considers scale-free network topology in the simulations.

**Figure 3:**
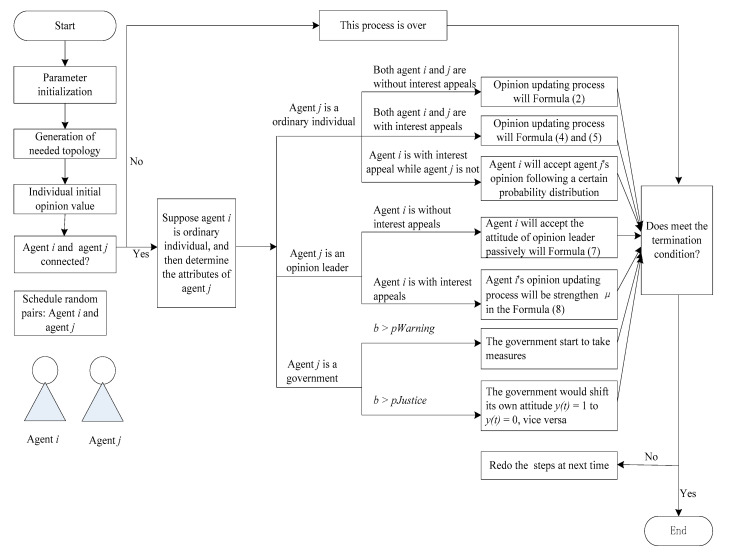
Multi-agent simulation processes.

Based on this kind of typology, we employ the random sampling method in the interactions. At every time step, two agents are randomly selected from all agents to justify whether they are connected according to the network adjacency matrix, and to which type it belongs according to agent attributes. The different agents interact with each other based on the rules above when they are connected. If the agents are not connected, the process will change to the next evolutionary time step. The process is repeated before the system becomes steady, and the impact of heterogeneous features on the structure reversal of public opinion of NIMBY conflict event is measured by steady state and steady time of the social system. The process framework of simulation experiment design is shown as follows (Figure 3).

### 4.2. Parameter Interpretation

The group scale has a significant impact on the activity as one of elements of collective behavior in 52 mass incidents occurred in China during 2007–2011 [55], as well as collective opinion evolution [48]; these can show that formation and evolution of any public opinion need a considerable number of participants. Meanwhile, the average scale of these 52 mass incidents was within the interval [100, 900], so in our simulation the size of the considered network is selected as *n* = 500. However, the mass incidents are generally triggered by a small group of citizens whose interests related to survival might be damaged, especially economic interests, which can be regarded as the seed agents in the initial stage of a NIMBY case. In this paper, the parameter is set to *nStart* = 5 to denote the number of seed individuals, they will further infect other agents. For all of the computer experiments, the ABM simulation is conducted 5000 iterations (or steps) with Netlogo 6.0.2 version. All simulation results are averaged 100 runs. The tested default parameters of the simulations are summarized in Table 1. These parameters are applied throughout all the simulations unless explicitly specified otherwise.

- To capture the heterogeneity of bigotry degree caused by different interest appeals of agents, let convergence parameter *μ* = 0.15 for the interaction between individuals with interests, while *μ* = 0.5 for the interaction between individuals with and without interests;
- The confidence level *d*_1_ for the interaction between the ordinary agents is supposed to equal 0.3, while *d*_2_ = 0.5 for the interaction between the ordinary agent and opinion leader;
- The thresholds of response and compromise of the government agencies are, respectively, *pWarning* = 0.2, and *pJustice* = 0.5;
- Collective actions occurred in China currently are caused by damage to the interests of the groups [56], which indicates that there are contradictions between the attitudes of the individuals and the government, especially the government often supports a certain issue further to damage the individuals’ interests. Thus, initial attitude of government agency is *y* (0) = 1 in our simulations.

## 5. Simulation Results and Discussion

### 5.1. Simulation Results of Default Parameters

**Figure 4:**
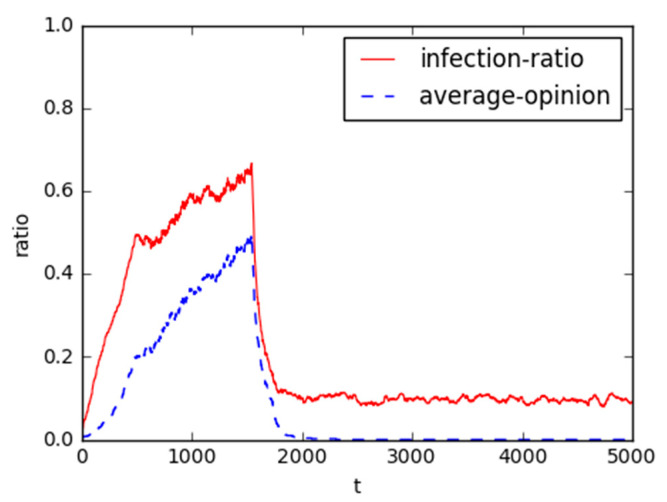
The results with the default parameters. It is the simulation results by using the value of parameters in Table 1. The rate of infected agents and the average opinion of all individuals are marked with red and blue, respectively. The meanings of these two lines are the same in the figures below.

Based on proposed models, the following simulation experiments are designed to analyze the interactive process among the individuals and the government agents in a social network, in order to understand the impact of multiple heterogeneities on the structure reversal of public opinion of NIMBY mass incident. Figure 4 is the result with the default parameters, and simulation experiments demonstrate that there is indeed an identifiable probability for online structure reversal effect.

Intuitively, as the average opinion (*b*) of groups taking part in mass incidents increases, the infection-ratio of the opinion followers is gradually increasing. When it increases to 0.2, the infection-ratio begins to fluctuate, and it indicates that there is a certain governance performance after the corresponding government responds to this incident, while the conflict of interest appeals cannot completely be resolved and the guiding power of the government decreases. Accordingly, when it reaches the peak, *b* is larger than *pJustice* = 0.5; the attitude of government changes from 1 (support) to 0 (compromise). This is the phenomenon of online public opinion structure reversal in mass incident that our research wants to unfold. Also, it can be observed that parameter *b* rapidly reduces until it closes to 0, this implies that it realizes the opinion consensus in the social network.

With the evolution of public opinion about Maoming PX event (located in Guangdong Province, China) as a case study [1], an opinion leader regarded as a seed agent posted a message about the event at 3:00 PM on 31 March 2014. This message made a comment on the anti-PX protest, which attracted a large number of followers, and triggered heated discussion on the risk of PX project. On 3 April, the Maoming government responded that “the PX project would never be started unless the society reach a full consensus” in a press conference. This indicated that the event was pacified because the government, in a strong position, compromised with the vulnerable groups through policy adjustment during the evolution of the event. It can be supported that there is a clear structure reversal of online public opinion for the interest conflict mass incident.

Specifically, users in the evolution process were classified into three groups in Maoming’s PX event: individuals, opinion leaders, and government agencies [1]. As for many followers in the Maoming anti-PX protest, most of them were ordinary netizens without any interest, they just posted and reposted messages frequently, but their personal influence power was rather limited. With the spread of posts in the Community, Weibo, WeChat and other platforms, their average opinion value increased across the rising up of online public opinions. At the same time, some local residents nearby the facilities location, the direct stakeholders, were the main participants in the conflict. The greater their risk perceptions of the project, the more likely they were to infect local residents to participate in the conflict event (i.e., the infection percentage of public opinion gradually increases), which in turn enhanced the average view value of participants.

In this case, “News Organizations” and “Elites” were the most active opinion leaders and highly involved in all stages [1], which catered to public attention and were more likely to evoke emotional resonance. In the formation cycle of public opinion, “News Organizations” got the “harmful or harmless” debate over the risks of PX production and reported the progress of the relevant news, leading to the surge of the risk perception. However, “the edit war”, “Elites” promoted the propagation of the emergency in the diffusion stage, magnifying the public’s “PX fear”. Subsequently, they were more likely to control the discourse, and set specific topic so as to guide the spreading direction of public opinion, which accelerated the offline and online behaviors of individuals.

When PX project triggered a violent clash by a handful of lawbreakers, from March 31 the government agencies (e.g., police station, municipal government) began to respond to it through face-to-face communication, television speeches, official announcements, press conferences, decision argumentation. and letters to citizens. Thus, the number of risk perception frame slowed down while the progress or solution frame continued to fast spread, which indicated the conflict between the local residents and the government agencies gradually began to ease. However, most citizens still expressed their dissatisfaction until the government shifted from initial support to final compromise. This evidenced the effect of governmental communication on the structure reversal process in the online public opinion of mass incident.

### 5.2. Influence of Heterogeneity of Health Interest Attribute

**Figure 5:**
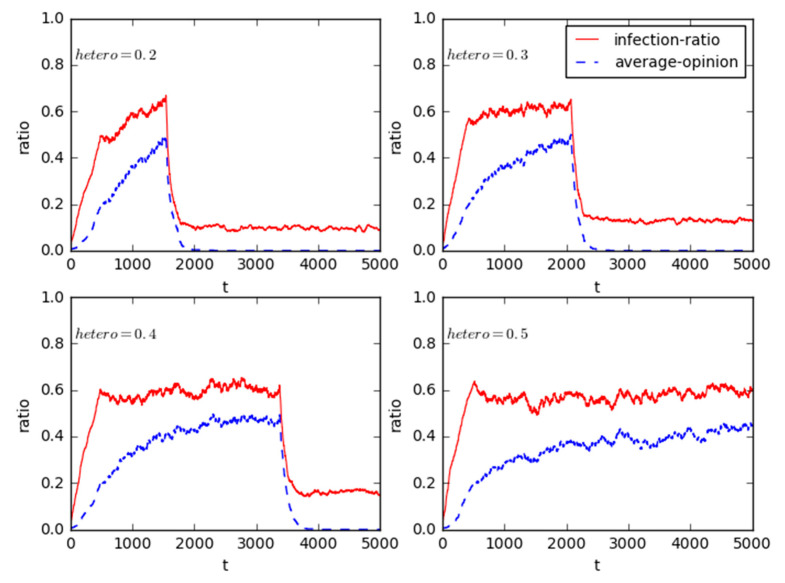
Sensitivity analysis for the heterogeneity of interest attribute.

Without loss of generality, we alter the proportion of individuals without health interest appeals to observe the impact of such changes on the online public opinion structure reversal in NIMBY event and influence power of the heterogeneous interest attributes. In this experiment, parameter *hetero* increases from 0.2 to 0.3, 0.4 and 0.5; Figure 5 illustrates the evolution results.

Figure 5 directly demonstrates that with increases in the proportion of agents with no health interest appeals, the dissemination speed of public opinion was slowed while the infection-ratio is still increasing, and the time node of online structure reversal also has been postponing. It can safely infer that the fraction of agents with interest appeals will play a great role in the online structure reversal, which can further support the proposition that the appeal of interests related to survival to the group is a main force for the online structure reversal in mass events.

More importantly, after *hetero* exceeds 0.5 the infection-ration fluctuates around 60%, although many individual agents were infected by the participants without health interest appeals and the purpose of network mobilization is also achieved, the average opinion of entire groups fails to reach the reversal threshold of government’s attitude because it is unable to realize the opinion consensus of the whole social network, so the structure reversal phenomenon does not occur. In fact, as illustrated in Figure 5, there is an upper threshold of *hetero* between 0.4 and 0.5 for the online public opinion structure reversal in mass incidents. Subsequently, we can infer that the individuals who want to express interests effectively through public opinion should mobilize the positive targets who also have similar interest appeals, especially interests related to survival. On the contrary, if the government will reasonably increase the ratio of citizens without interest appeals, the public opinion can be controlled, as well structure reversal will not take place.

### 5.3. Influence of Heterogeneity of Opinion Leaders

Without any change in other conditions, we just vary the value of *nLeader* from 20 to 30, 40, and 50.

**Figure 6:**
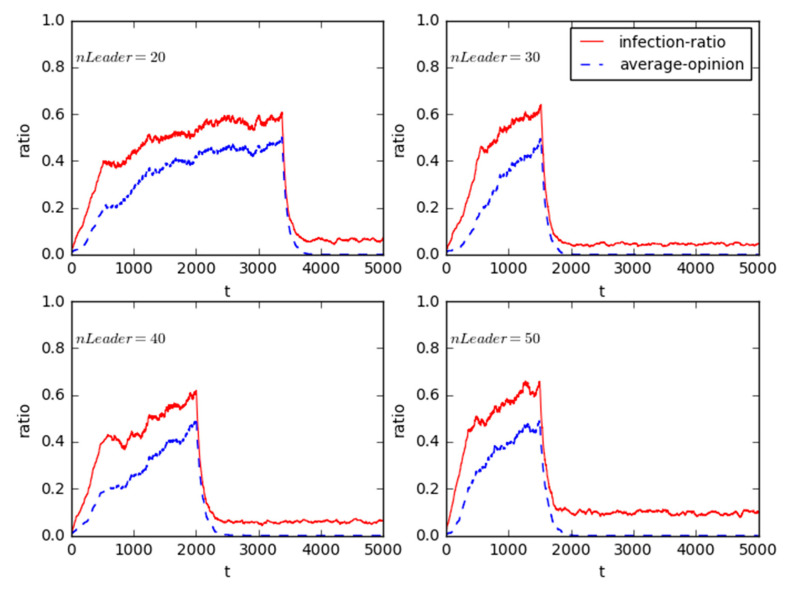
Sensitivity analysis for the heterogeneity of opinion leader’s amount.

As shown in Figure 6, with the increase of total number of opinion leaders, in the mass incidents the infection-ratio becomes much higher, and this also makes the time much earlier step by step when the online public opinion structure reversal occurs. This provides enough evidence to conclude that in a mass incident the more the opinion leaders involve, the quicker the public opinion disseminates, which is consistent with many existing research results [56]. Since the individuals with direct interests in the collective actions often tend to be weakly organized and lack strong organizational connections, most of them do not have a clear action plan. Moreover, the citizens without health interest appeals are numerous, but they fail to have the common organizational identities and coherent social positions, and lack necessary links with each other, which causes their participations with different depths and durations. Accordingly, after the involvements of public opinion leaders (e.g., grassroots elites, public intellectuals, celebrities, etc.) who are keen on public affairs and have strong public spirits, they can write and spread comments, blogs, or even take practical actions to make the people with interests and others without interests be closely linked together, and this will put much more pressure on the corresponding government and force it to compromise eventually. This also addresses and supports the influence of opinion leaders of media [57].

Intuitively, one may argue that the guiding powers of the opinion leaders increase with the number of the opinion leaders in a fixed-size group. However, as illustrated in Figure 6, we can also clearly find that the influence of the leaders on the followers begins to become weak in the whole after the number of leaders is larger than 30. That is to say, the opinion leaders fraction exceeds 6% (30/500 = 6%) in the online public opinion structure reversal of NIMBY mass incidents, then the guiding powers of the opinion leaders will be unchanged, which is an important finding in our research and is somewhat confirmable with that in [56].

### 5.4. Influence of Heterogeneity of Government’s Ability

#### 5.4.1. Influence of the Government’s Response Threshold

**Figure 7:**
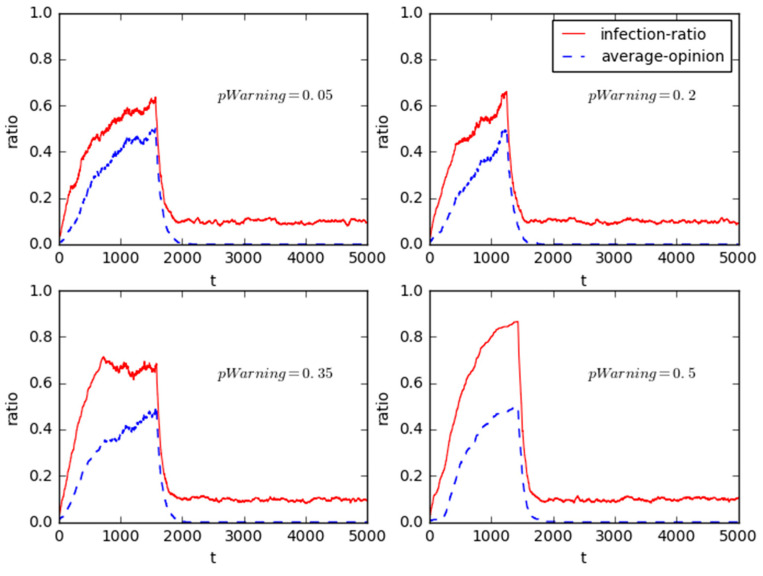
Sensitivity analysis for the heterogeneity of government response threshold.

As shown in Figure 7, four experiments are done when the value of *pWarning* are shifted from 0.05 to 0.2, 0.35, and 0.5, respectively.

Additionally, the result also shows that the convergence times of public opinion structure reversal are not varied over the values of *pWarning*. One possible reason is the measures taken by the corresponding government are difficult to inhibit the growth of collective opinions in a timely manner due to the much higher public opinion dissemination speed. Another reason may be related to the passive response of the government. In the mass incidents, in order to make public opinion favorable to itself, the government will usually stand on the opposite side of the public, and take some rigidly controllable countermeasures to deal with the public opinion negatively, such as refusing appeals and dialogues, controlling media, blocking news, violence conflict, or keeping silent and not disclosing any information about the event, etc.; these ways would be bound to accelerate the public opinion of mass incident. In fact, a series of anti-PX protests occurred in Xiamen (2007), Dalian (2011), Ningbo (2012), Chengdu (2013), Kunming (2013), Maoming (2014) and Shanghai (2015) in China [58], through investigating these incidents it could be found that the government agencies did not disappear to intervene in the communicate messages with the stakeholders in the first place, while they had to passively make official announcements on news organizations (e.g., Daily newspaper, Weibo) to keep the public informed in order to quell the interest conflict events after it was likely that there had been a flurry of discussing on mass media. Besides, most of them kept silent in the second propagation cycle.

#### 5.4.2. Influence of the Government’s Reversal Threshold

To observe the influence of judgment threshold with compromise or not on the online public opinion structure reversal in NIMBY case; in this experiment, the *pJustice* value of government’s reversal threshold is initially set as 0.5, accordingly, *pJustice* is followed to increase to 0.55, 0.6, 0.65, and 0.7, respectively.

**Figure 8:**
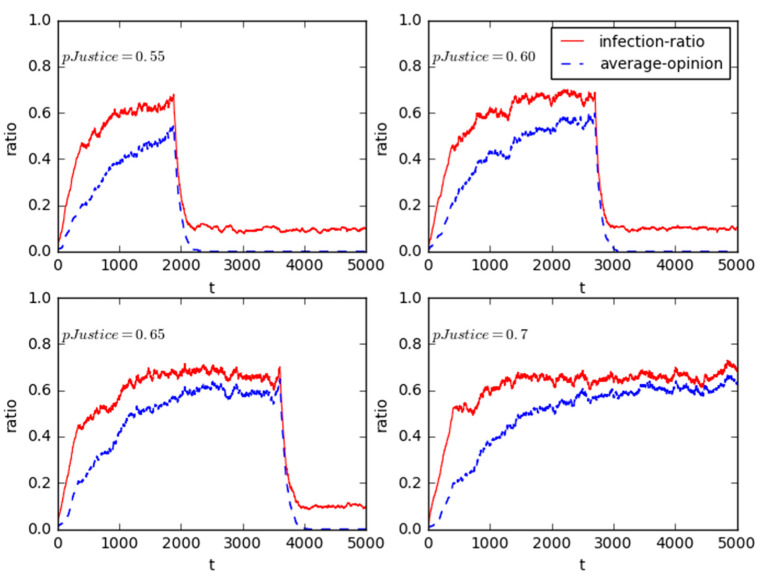
Sensitivity analysis for the heterogeneity of government reversal threshold.

The results in Figure 8 illustrate the structure reversal processes of online public opinion, these represent the evolution trajectories of infection-ratio and average opinion over time, respectively. Regardless of the reversal thresholds of government (0.5, 0.55, 0.60, 0.65, and even 0.7), both infection-ratio and average opinion can be converged. However, as the increase of the government’s reversal threshold, the opinion evolution relatively requires more time to reach a stable state. This addresses the important role of government’s reversal threshold in delaying the time when online public opinion structure reversal occurs. Furthermore, the higher the threshold of government’s compromise is, the much stronger emergency response ability the involved government agency has.

As for government agencies involved in PX events, they preferred to interact with news media and were less likely to interact with the individuals in the responsive process as government agencies had a high degree of authority. Moreover, comparing PX event in Xiamen to Maoming, it was clear that the former lasted for more than nine months, while the latter was just 4 days. It is because that except for the involvement of the municipal government and its agencies in the Maoming, there were some superior sectors in the government involved in Xiamen, including provincial government and Ministry of Environmental Protection, which impacted their actual communication effectiveness. Certainly, we always believe that different levels of government agencies have different governance abilities and trust degrees, these heterogeneities are bound to result in different “compromise” judgment thresholds.

## 6. Conclusions

Based on the classic dynamics model, this paper then considers the differences in both the interactions between the public with and without public health interest appeals and the interactions between the government and the individual agents to propose a novel opinion dynamics model, and uses an agent-based simulation technique to investigate the influence power of heterogeneity in the interest attribute, the number of opinion leaders, and abilities of a government response to online public opinion. In summary, through the comparative analysis of simulation experiments, we can conclude that these heterogeneities played much more important roles on structure reversal of online public opinion in a given mass event.

(1) The proportion of individual agents without health interest appeals will delay the online public opinion structure reversal. Besides, when this ratio is between 0.4 and 0.5, there is an upper threshold to cause the online public opinion structure reversal.
(2) Our study also confirmed the great role of opinion leaders in the structure reversal process of online public opinion. However, as the increase of opinion leaders in size or fraction, although the occurrence time of structure reversal could become much earlier, their impacts are of no significant differences for both the public opinion dissemination spread and the time of structure reversal when it increased to about 6%. In short, we can never neglect the importance of opinion leaders, so governments should pay more attention to the size of opinion leaders when they monitor and give early warnings, or respond the public opinion in mass incidents. Certainly, the corresponding government agencies could interact with news media to form a group, acting as the opinion leaders, which can effectively capture public attention and avoid activating a new opinion topic.
(3) The double threshold effects of the government’s response to online structure reversal were confirmed. During the public opinion process, the number of individuals without interests will vary over the increase of the citizens with interests, this could force the corresponding government to passively take measures to respond negatively at the beginning. Accordingly, due to the illusory truth effect, the true or false news about this incident would continue to ferment and ignite the emotions of the cyber users everywhere, which in turn forcedly requires the government to timely take effective measures to appease the individual’s sentiments. However, this research notes that online public opinion structure reversal depends on two crucial factors: one is the proportion of the individuals with interests and the other is the government’s response thresholds.

Although the simulation results of this study can shed new lights on the process of online public opinion structure reversal, there are still some limitations. First, it may be difficult to reflect the objective reality in the social system by randomly assigning some agents as opinion leader nodes in our study. Future work can apply the centrality measures or a new measure to detect opinion leaders. Besides, this research just considers the total number of opinion leaders, while there are more than two subgroups of opinion leaders in a given social network, such as positive or negative opinion tendency. In subsequent work, we plan to further investigate their differences in the target opinions held by these subgroups and their effects in online public opinion structure reversal. Second, we carried out the experiments on one certain dimension, but generally an opinion of the public includes several dimensions. We are sure that it is more interesting to consider the cases in which specific agents with multidimensional distribution in the social networks. In the future, we can continue to predict the structure reversal probability of online public opinion in mass incidents with dynamic structure. Third, to simplify the study, the default values of some parameters in our experiments were chosen according to the related works, especially convergence parameter *μ* and bounded confidence level *d*, these parameters are needed to further test in the future. Finally, this research just stated that in different scenarios, the government measures response to NIMBY conflict cases can differ, while it is not clear that how are they implemented. In the future, we can further explore how fail how the measures are implemented in the simulations and the model, and what are the dynamics that determine the government’s attitude, etc.

## Figures and Tables

**Table 1:** Default simulation parameters.

Parameter	Meaning	Default
*n*	Total number of individuals	500
*nStart*	Number of seed agents in the initial stage	5
*nLeader*	Number of opinion leaders	50
*hetero*	Fraction of individuals without interest appeals	0.2
*u*	Convergence parameter for the interaction between individuals with interests	0.15
	Convergence parameter for the interaction between individuals with and without interests	0.50
*d*_1_	Bounded confidence level between the ordinary agents	0.5
*d*_2_	Bounded confidence level between the ordinary agent and opinion leader	0.3
*ε*	Generated random value	Random (0, 0.5]
*pWarning*	Response threshold of government	0.2
*pJustice*	Reversal threshold of government’s attitude	0.5
*x* (0)	Initial opinion value of individual agent	Random [0, 1]
*y* (0)	Initial attitude of the government agency	1
